# Acute death of astrocytes in blast-exposed rat organotypic hippocampal slice cultures

**DOI:** 10.1371/journal.pone.0173167

**Published:** 2017-03-06

**Authors:** Anna P. Miller, Alok S. Shah, Brandy V. Aperi, Shekar N. Kurpad, Brian D. Stemper, Aleksandra Glavaski-Joksimovic

**Affiliations:** 1 Department of Neurosurgery, Medical College of Wisconsin, Milwaukee, Wisconsin, United States of America; 2 Department of Cell Biology, Neurobiology & Anatomy, Medical College of Wisconsin, Milwaukee, Wisconsin, United States of America; 3 Clement J. Zablocki Veterans Affairs Medical Center, Milwaukee, Wisconsin, United States of America; University of Florida, UNITED STATES

## Abstract

Blast traumatic brain injury (bTBI) affects civilians, soldiers, and veterans worldwide and presents significant health concerns. The mechanisms of neurodegeneration following bTBI remain elusive and current therapies are largely ineffective. It is important to better characterize blast-evoked cellular changes and underlying mechanisms in order to develop more effective therapies. In the present study, our group utilized rat organotypic hippocampal slice cultures (OHCs) as an *in vitro* system to model bTBI. OHCs were exposed to either 138 ± 22 kPa (low) or 273 ± 23 kPa (high) overpressures using an open-ended helium-driven shock tube, or were assigned to sham control group. At 2 hours (h) following injury, we have characterized the astrocytic response to a blast overpressure. Immunostaining against the astrocytic marker glial fibrillary acidic protein (GFAP) revealed acute shearing and morphological changes in astrocytes, including clasmatodendrosis. Moreover, overlap of GFAP immunostaining and propidium iodide (PI) indicated astrocytic death. Quantification of the number of dead astrocytes per counting area in the hippocampal cornu Ammonis 1 region (CA1), demonstrated a significant increase in dead astrocytes in the low- and high-blast, compared to sham control OHCs. However only a small number of GFAP-expressing astrocytes were co-labeled with the apoptotic marker Annexin V, suggesting necrosis as the primary type of cell death in the acute phase following blast exposure. Moreover, western blot analyses revealed calpain mediated breakdown of GFAP. The dextran exclusion additionally indicated membrane disruption as a potential mechanism of acute astrocytic death. Furthermore, although blast exposure did not evoke significant changes in glutamate transporter 1 (GLT-1) expression, loss of GLT-1-expressing astrocytes suggests dysregulation of glutamate uptake following injury. Our data illustrate the profound effect of blast overpressure on astrocytes in OHCs at 2 h following injury and suggest increased calpain activity and membrane disruption as potential underlying mechanisms.

## Introduction

The rate of blast-induced traumatic brain injury (bTBI) has escalated among active duty military personnel and veterans involved in recent military campaigns [1–4]. Symptoms of bTBI manifest on a scale of mild to severe and often involve physical, cognitive, emotional, and social deficits [5–10]. Moreover, a soldier’s reluctance to seek treatment [11], compounded with a potential misdiagnosis of post-traumatic stress disorder (PTSD) [3, 5] can impede recovery. Current treatment strategies are mainly focused on rehabilitation, mental health services, and symptom amelioration [12]. However, there is no available therapy that can stop or reverse the neurodegenerative cascade that follows primary cell death caused by blast exposure. Moreover, mechanisms underlying early and delayed cell death following bTBI remain elusive. Preclinical and clinical data suggest different underlying mechanisms and injury manifestations between blunt TBI and bTBI [13–16]. For these reasons, answering fundamental questions regarding bTBI neuropathology is prerequisite for the development of more effective therapy protocols. Specifically, it is necessary to assess early cellular and molecular changes following bTBI to establish potential therapeutic strategies to prevent or ameliorate the spread of neurodegeneration.

Direct effects of blast exposure on brain tissue remain controversial. It has been proposed that blast overpressure indirectly causes brain injury either via skull deformation, head acceleration, ischemia, or thoracic mechanisms [17–23]. However, research from our group, in addition to the results of other experts in the field, suggests that a blast shock wave can transverse the cranium intact and generate tissue stress and strain leading to neuronal damage [24–29]. Correspondingly, data from *in vitro* bTBI models [30–33], including our recent findings [34], imply that blast overpressure can directly damage neurons and glial cells. In previous rat bTBI studies conducted by our [16, 28] and other groups [19, 35, 36], exposure to the peak overpressure magnitudes in the range of 100 to 450 kPa resulted in neurodegenerative changes and behavioral impairments. Likewise, exposure of OHCs to the blast overpressures of about 150 (low) and 280 kPa (high) in our previous [34] and present *in vitro* studies evoked significant and progressive cell death, confirming validity of our test conditions.

Neurodegenerative disorders are traditionally investigated with a neuron-centric approach, but it is becoming increasingly recognized that glial cells, including astrocytes, are implicated in neurodegenerative disorders and brain injury [37–41]. Under normal physiological conditions, astrocytes play a pivotal role in maintenance of brain homeostasis through control over cerebral blood flow and metabolism, ionic spatial buffering, regulation of water, control of biosynthesis and turnover of amino acid neurotransmitters, and providing energy and nutrient support for neurons [42–47]. Astrocytes also have the ability to control synaptogenesis, integrate neuronal inputs, release a variety of transmitters, and modulate synaptic activity [48–54]. However, astrocytes are affected in many neurodegenerative disorders [55–59], and their altered function contributes to further spread of neurodegenerative changes [60–62]. Although the exact role of astrocytes in neurodegeneration is unknown, it is believed that different mechanisms such as change in glutamate uptake and release, activation of astrocytes, and their death may contribute to neuronal loss [37, 38, 58, 63, 64]. Changes in astrocytic functions and the above mechanisms have also been associated with TBI [40, 41, 65]. Though largely dependent on severity and mechanical properties of the injury, reactive astrogliosis has been observed following both blunt and bTBI [35, 40, 66–69]. Spectrum of morphological, molecular and functional changes that astrocytes undergo in reactive astrogliosis, also known as astrocytosis, include upregulation of glial fibrillary acidic protein (GFAP) and other intermediate filaments, hypertrophy of cell body and processes, and in more severe cases proliferation and scar formation [70]. Additionally, several animal [71–75] and human [76] non-blast TBI studies have indicated death of astrocytes by the decrease in the total number of cells labeled with the astrocytic marker GFAP, or by co-staining with GFAP and markers of apoptotic cell death including terminal deoxynucleotidyl transferase dUTP nick end labeling (TUNEL) and activated caspase 3. In human postmortem material obtained from cases with severe and complicated TBI, astrocytes that underwent an continuum of oncosis, apoptosis and necrosis were detected based on morphological changes such as swollen nucleoplasm, cytoplasm, and cell organelles, chromatin condensation and marginalization, formation of apoptotic bodies, and plasma membrane fragmentation [77]. Two recent studies also reported presence of dead, TUNEL and GFAP co-labeled astrocytes in rat and monkey bTBI models [67, 78]. However, the exact role of astrocytic activation and death in the etiology of bTBI is unclear and the acute response of astrocytes to a blast overpressure is not well characterized. Therefore, our present study focuses on understanding how astrocyte morphology and viability are affected by a blast overpressure at an early time point following injury and to elucidate mechanisms underlying blast-evoked astrocytic death.

Our previous studies in *in vitro* bTBI model were mainly focused on characterizing the neuronal and glial response to blast exposure at 72 hours (h) post-injury [34]. At this time point, we demonstrated significant neuronal loss and robust activation of astrocytes and microglial cells in OHCs exposed to blast overpressure [34]. While only a small number of dead glial cells were present at 72 h post-injury, dead microglial cells were observed at 4 and 24 h post injury [34]. The aim of the current study was to characterize the acute astrocytic response to a blast overpressure exposure using the same *in vitro* bTBI model. As done previously in studies utilizing OHCs [79–81], we have visualized astrocytes using immunostaning against GFAP that is expressed in the large portion of heterogeneous population of astrocytes [70, 82]. Additionally, co-labeling of GFAP with propidium iodide (PI) or Annexin V was used to assess necrotic or apoptotic astrocytic death, respectively. To further study mechanisms that lead to the early blast-evoked astrocytic death, immunohistochemical staining, western blot, and a dextran permeability assay were conducted. Contrary to our previous data collected in *in vitro* bTBI model at 72 h post-injury, present study demonstrated significant number of dead, necrotic astrocytes at 2 h post-injury, suggesting different response of astrocytes at the acute and the later phase of bTBI. An understanding of different temporal effects of blast exposure on astrocytes is of the particular interest in illuminating mechanisms that lead to the spread of neurodegeneration and development of more effective therapies for bTBI.

## Methods

### Animals

Time-pregnant, Sprague Dawley (SD) rats (Charles River Laboratories, Wilmington, MA, USA) were housed in individual cages, with standard colony conditions, until parturition. Food and water were available *ad libitum*. Hippocampal tissue used to prepare OHCs was obtained from postnatal rats (P7-P10; n = 59). All laboratory animal procedures were completed in congruence with the National Institutes of Health (NIH) Guide for the Care and Use of Laboratory Animals and approved by the Zablocki Veterans Affairs Subcommittee for Animal Studies (Protocol number: 3171–01).

### Preparation of OHCs

OHCs were prepared under aseptic conditions using a modified procedure from Stoppini and colleagues [34, 83]. Postnatal rats were euthanized via decapitation and the brains were removed via a longitudinal cut along the midline of the skull. The hippocampi were isolated in cold dissecting medium (pH 7.2) which was composed of 50% Minimum Essential Medium (MEM), 50% calcium and magnesium free Hanks Balanced Salt Solution (HBSS), 20 mM HEPES (N-2- hydroxyethylpiperazine-N’-2-ethanesulfonic acid), 7.5 g/l D-glucose, and 1% Penicillin/Streptomycin (all obtained from ThermoFisher Scientific, Grand Island, NY, USA) [34, 84]. Using a McIlwain tissue chopper (Ted Pella, Inc., Redding, CA, USA) hippocampi were cut transversely into 400 μm-thick sections. Hippocampal sections were separated under a dissecting microscope with sterile spatulas and inspected for intact morphology. Only intact sections were transferred to 0.4 μm porous MilliCell cell culture inserts (EMD Millipore, Billerica, MA, USA) and grown in 6-well plates containing 1 ml of serum-based media consisting of 50% MEM- Hanks medium, 25% HBSS, 25% horse serum, 50 mM HEPES, 2 mM L-glutamine, 5 mg/ml D glucose, and 1% antibiotic/antimycotic (all obtained from ThermoFisher Scientific except horse serum which was obtained from Atlanta Biologicals, Flowery Branch, GA, USA) [34, 85–87]. OHCs were maintained at 37°C with 5% CO_2_ for the duration of the studies.

One day following dissection, the culture media was replaced with fresh serum-based media. From 4 to 7 days *in vitro* (DIV), the serum-based media was gradually changed to serum-free media consisting of 50% MEM- Hanks, 25% HBSS, 25% Neurobasal-A medium, 17 mM HEPES, 2 mM L-Glutamine, 2% B-27, 5 mg/ml D-glucose, and 1% antibiotic/antimycotic (all obtained from ThermoFisher Scientific). Starting from the 7^th^ DIV until the end of experiment, OHCs were maintained in the serum-free media [34, 86, 88] in order to decrease glial activation, proliferation and scar formation [79, 89]. As described below, PI uptake measurements were also performed in serum-free media as DNase I present in the serum can degrade the DNA of dead cells and lead to an inaccurate estimate of the number of dead cells [90].

### Blast injury of OHCs with an open-ended helium-driven shock tube

OHCs were grown for 8 days in culture prior to blast exposure. As previously demonstrated by data from our and other laboratories, this period is sufficient to allow recovery from any procedure-related degeneration [34, 91, 92]. An open-ended, helium-driven shock tube, built by our group, was used to generate overpressures of specific magnitudes and to produce injury in OHCs as described previously [34]. Briefly, individual membrane inserts containing OHCs were placed in 40-mm culture dishes with 800 μl of serum-free media. Culture dishes were next covered with Parafilm and sealed within sterile plastic pouches (5.5 cm x 6.5 cm). OHCs were placed on a rigid stand 22 cm away from the end of the shock tube and were positioned 55° off axis to avoid exposure to exhaust gases. OHCs were injured using a single blast overpressure designated as either low (138 ± 22 kPa) or high (273 ± 23 kPa), since our previous studies demonstrated that exposure to overpressures of about 150 kPa and 280 kPa results in significant cell death at 2 h following injury [34]. Sham control OHCs were processed identical to blast-injured OHCs, but were not exposed to the blast overpressure.

### Immunohistochemistry

At 2 h post injury, OHCs were fixed for 30 minutes (min) at room temperature (RT) with 4% paraformaldehyde (PFA) in 0.1 M phosphate buffer (PB) and immunohistochemistry (IHC) was performed [34]. At the time of fixation, OHCs were about 100 μm thick as they get thinner after a few days in culture [93–95]. Immunostaining with a polyclonal rabbit antibody against GFAP (Dako, Carpinteria, CA, USA Cat# Z0334, RRID: AB_2314535) was conducted directly on the insert membrane, which was cut out around the OHCs [34, 94, 96]. Additionally, OHCs that were pretreated with either Annexin V conjugated to Alexa 488 (ThermoFisher Scientific) or 10,000 kDa Dextran (Dex10) conjugated to Alexa 488 (ThermoFisher Scientific), as described below, were gently peeled from the membrane insert and immunostained against GFAP (Dako) using the free-floating sections method [97]. Double labeling against GFAP (Dako) and glial glutamate transporter 1 (GLT-1; EMD Millipore Cat# AB1783, RRID: AB_90949) was also performed on the free-floating OHCs. Both OHCs attached to the insert membrane and free-floating sections were immunostained using the same protocol. Briefly, fixed OHCs were washed 3 times for 5 min in phosphate buffered saline (PBS) and incubated for 1 h at RT in blocking solution consisting of PBS, 5% normal goat serum (NGS; ThermoFisher Scientific), 5% bovine serum albumin (BSA; Sigma Aldrich, St. Louis, MO, USA) and 1% Triton-X 100 (TX-100; Sigma Aldrich). Following blocking, OHCs were incubated in GFAP (Dako) primary antibody diluted in blocking solution at 1:500 for 48 h at 4°C in a humid atmosphere. For double staining, under the same conditions OHCs were simultaneously incubated with polyclonal rabbit antibody against GFAP (Dako; 1:500) and polyclonal guinea pig antibody against GLT-1 (EMD Millipore; 1:1500). After washing, OHCs were incubated with appropriate secondary antibodies diluted in blocking solution without TX-100 as follows: Alexa Fluor 488 conjugated polyclonal goat anti-rabbit antibody (ThermoFisher Scientific Cat# A-11034, RRID:AB_2534092; 1:500), Alexa Fluor 647 conjugated polyclonal goat anti-rabbit antibody (ThermoFisher Scientific Cat# A-21245, RRID:AB_2535813; 1:750), and Alexa Fluor 488 conjugated polyclonal goat anti-guinea pig antibody (ThermoFisher Scientific Cat# A-11073, RRID:AB_2534117; 1:400). Staining specificity was confirmed by omission of primary antibody. Sections were counterstained with the nuclear dye DAPI (4’, 6-diamindino-2-phenylindole dihydrochloride; Sigma-Aldrich) and mounted with VECTASHIELD HardSet mounting medium (Vector Laboratories, Burlingame, CA, USA). Immunostained OHCs were analyzed using a Leica TCS SP8 confocal laser scanning microscope (Leica Microsystems, Buffalo Grove, IL, USA).

### Quantification of dead astrocytes

The red fluorescent dye, PI (ThermoFisher Scientific) was used to assess cell death following blast injury [34, 98]. PI enters only into cells with a damaged cell membrane and is considered to be primarily a marker of necrotic cells [99]. OHCs were placed in media containing 2 μM PI [100, 101] for 2 h prior to imaging. All sections were imaged under identical conditions at prior to (basal) and 2 h following blast injury using a Nikon Eclipse TE2000-U upright fluorescent microscope (Nikon Instrument Inc., Melville, NY, USA) equipped with a digital SPOT camera and software (Spot Imaging Solutions, Sterling Heights, MI, USA). Any OHC which had dissection or handling related damage prior to the beginning the experiment was excluded [84, 87, 88, 100]. Following PI imaging at 2 h post-injury, OHCs were fixed and processed for IHC as described above.

GFAP immunostained OHCs were used to acquire three non-overlapping images (290.63 μm by 290.63 μm) of the cornu Ammonis 1 (CA1) region by a Leica TCS SP8 confocal laser scanning microscope, and only a small fraction of CA1 region was not analyzed [34, 80]. Two observers blinded to the experimental groups counted in each image the total number of dead astrocytes that were co-labeled for GFAP and PI. Counts obtained from the two observers were averaged and expressed as the total number of dead astrocytes per counting region [34]. The concordance correlation coefficient (CCC) was used to evaluate agreement among two observers.

### Apoptosis assay

Annexin V binds with high affinity to membrane phospholipid phosphatidylserine (PS), which is transposed to the external side of the plasma membrane during early apoptosis [99, 102]. Accordingly, in our study Annexin V was used to assess the presence of apoptotic cells in OHCs in the acute phase of blast injury. Immediately following blast exposure, Annexin V conjugated to Alexa Fluor 488 (ThermoFisher Scientific Dead Cell Apoptosis Kit; 15 μl/ml) was added to the culture medium of control and OHCs exposed to blast overpressure. Simultaneously, OHCs were treated with PI (EMD Millipore; 2 μM). Following a 2 h incubation with Annexin V and PI, OHCs were fixed for 30 min at RT with 4% PFA in 0.1 M PB and immunostained against GFAP as described above.

### Calpeptin treatment of OHCs

The calpain inhibitor calpeptin (Santa Cruz, Dallas, Texas, USA; 10 μM) [103] was added to the OHCs’ serum-free medium followed immediately by a sham- or blast-injury. After the injury, samples were transferred to fresh serum-free medium containing the same concentration of calpeptin and PI (EMD Millipore; 2 μM). PI stained OHCs were imaged at 2 h post-injury and subsequently harvested for GFAP western blot analysis as described below.

### Gel electrophoresis, western blot, and densitometry

For western blot analysis, 10 OHCs from two different inserts of the same experimental group were pooled to represent one sample [104]. Tissue was lysed in 300 μl lysis buffer consisting of 50 mM Tris-HCl, 150 mM NaCl, 1 mM ethylenediaminetetraacetic acid (EDTA), 0.1% sodium dodecyl sulfate (SDS), 1% Triton X-100, and 1% mammalian protease and phosphatase inhibitor cocktail [105] (All obtained from ThermoFisher Scientific, except for SDS which was obtained from Bio-Rad, Hercules, CA, USA, and Triton X-100 which was obtained from Sigma-Aldrich). Slice homogenates were prepared using a manual homogenizer (ThermoFisher Scientific), incubated on ice for 30 min, and were further centrifuged at 13,000 RPM for 20 min at 4°C. The supernatant was collected and stored at -80°C until use.

The protein concentration was determined by colorimetric detection with a bicinchoninic acid (BCA) assay (ThermoFisher Scientific) according to the manufacturer’s protocol. Briefly, 10 μl of protein sample, or BSA standard, was loaded into a microplate. 200 μl of working solution was added to each sample and the microplate was incubated for 30 min at 37°C. The absorbance was measured at 562 nm using a microplate reader (PowerWave XS, BioTek Instruments Inc., Winooski, VT).

For gel electrophoresis, samples were heated with laemmli Sample Buffer (Bio-Rad) and 100 mM of dithiothreitol (DTT; MidSci, Saint Louis, MO, USA) at 95°C for 5 min. 15 μg/μl of sample protein was loaded onto a 10% Mini-PROTEAN TGX Stain-Free Precast Gel (Bio-Rad) and run under reducing conditions. The gel was blotted onto a polyvinylidene fluoride (PVDF) Immobilon ^®^ FL membrane (EMD Millipore) via wet transfer. Following a brief wash in tris-buffered saline (TBS; pH 7.5), the membrane was blocked with 5% BSA in TBS supplemented with 0.05% Tween 20 (TTBS). Further, the membrane was probed with primary antibodies against GFAP (Dako; 1:10,000), calpain (Santa Cruz Cat# sc-373966, RRID: AB_10917913); 1:1,000) or GLT-1 (EMD Millipore; 1:20,000) at 4°C overnight. Following a brief wash, membranes were incubated with goat anti-rabbit (ThermoFisher Scientific Cat# 31460, RRID: AB_228341; 1:10,000), goat anti mouse (ThermoFisher Scientific Cat# 31430, RRID: AB_228307; 1:10,000) or goat anti-guinea pig (ThermoFisher Scientific Cat# A18769, RRID: AB_2535546; 1:50,000) poly-horse radish peroxidase (HRP) conjugated secondary antibody diluted in blocking solution, respectively. A monoclonal antibody against mouse glyceraldehyde-3-phosphate dehydrogenase (GAPDH; Advanced Immunochemical, Long Beach, CA USA Cat# 6C5; 1:50,000) was used as a loading control with a secondary goat anti-mouse (ThermoFisher Scientific; 1:50,000) HRP conjugated antibody. The membrane was then incubated with SuperSignal West Femto (ThermoFisher Scientific) for 5 min at RT and the signal was detected using a Carestream ECL 4000MM Pro imaging scanner (Carestream Inc., Rochester, NY, USA). Bands were scanned into digital images and analyzed by densitometry using Carestream Molecular Imaging Software Standard Edition v. 5.3.4.17821. Target band density was normalized to the appropriate loading control [106].

### Assessment of altered membrane integrity following bTBI

Cell membrane disruption following blast exposure was evaluated using Alexa 488-conjugated Dex10, which is excluded from cells with an intact cell membrane [107, 108]. OHCs were incubated with Alexa 488-conjugated Dex10 (ThermoFisher Scientific; 0.02 mg/ml) from 2 h before injury until 2 h post-injury. Immediately following injury, PI (EMD Millipore; 2 μM) was also added to the OHCs’ culture medium. Following imaging of PI staining at 2 h post-injury, OHCs were fixed and immunostained against GFAP as described above. To assess involvement of cell membrane perturbation in astrocytic cell death, co-labeling of GFAP, PI, and Dex10 was evaluated in mounted OHCs under TCS SP8 confocal laser scanning microscope (Leica Microsystems).

### Data collection and statistical analysis

Quantification of dead astrocytes for each experimental group was performed on 5 OHCs obtained from at least 3 different animals. Three images in the CA1 region per section were acquired and were subsequently used for quantification of GFAP and PI co-labeled cells. Data are presented as the total number of dead astrocytes per counting area in the CA1 ± standard error of mean (SEM).

Western blot analysis for each protein was repeated on samples from at least 3 different experiments, with each sample consisting of 10 OHCs. Quantification of GLT-1- and GFAP-immuoreactive bands was performed using computer-assisted densitometry scanning. Data collected from 3 different samples per group were averaged and presented as arbitrary densitometry units of GLT-1 or GFAP relative to GAPDH loading control.

Statistical comparison among groups was done by one way analysis of variance (ANOVA) followed by a Tukey’s *post hoc* test. A value of P < 0.05 was considered statistically significant.

## Results

### Acute astrocytic response to blast overpressure

**Fig 1:**
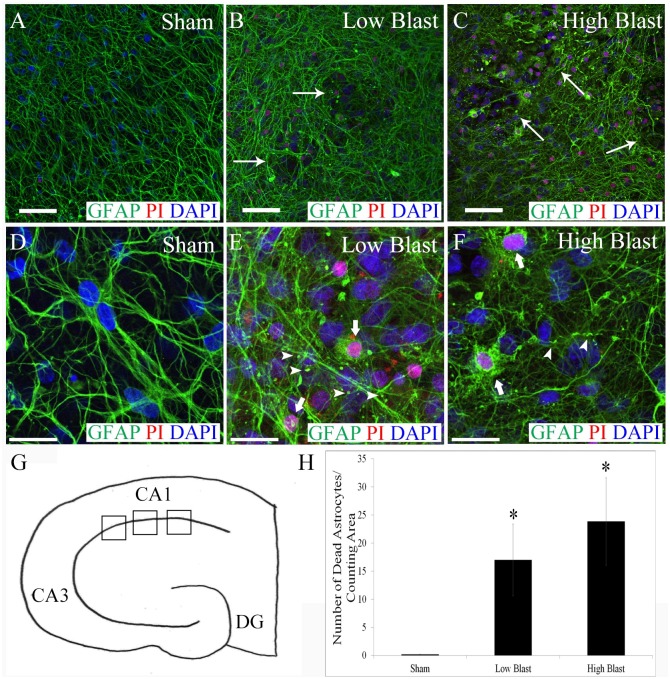
Acute morphological changes and demise of astrocytes following blast exposure. Representative confocal images acquired in the CA1 hippocampal region from sham controls (A, D), low-blast (B, E), and high-blast (C, F) OHCs that were fixed at 2 h following injury and stained with an anti-GFAP antibody (green), PI (red), and DAPI (blue). Shearing of the astrocytes (thin arrows) was detected in OHCs exposed to blast overpressure (B, C) while it was absent in the sham controls (A). Clasmatodendrosis (arrowheads) was also observed in the low- (E) and high-blast (F) groups, but it was very infrequent in the sham control group (D). At the same time point, only a few dead astrocytes were present in sham control OHCs (D) while significant number of dead astrocytes (thick arrows) was revealed in the low- (E) and high-blast (F) groups. (G) Schematic diagram of OHC, indicating approximate locations in the CA1 region (boxes) where images for quantification of dead astrocytes were taken. (H) Number of dead astrocytes per counting area in the CA1 hippocampal region at 2 h following injury was significantly higher in both the low- (*; P< 0.05; n = 5) and high-blast groups (*; P < 0.05; n = 5) compared to the sham control group (n = 5). Scale bars (A-C) 50 μm (D-F) 20 μm.

At 2 h following OHCs’ exposure to the blast overpressure, we have detected shearing, or tearing, of astrocytes as well as changes in their morphology, including swelling and beading, while astrocytes with mainly intact morphology were present in the sham control OHCs (Fig 1A–1C). In both the low- and the high-blast groups, we have observed clasmatodendrosis, which is defined by the beading and dissolution of astrocytic processes (Fig 1E and 1F). In OHCs exposed to a blast overpressure, clasmatodendrosis was present throughout the tissue (Fig 1E and 1F), whereas it was almost absent in the sham control group (Fig 1D). Moreover, at 2 h post-injury, we have observed in both the low- and high-blast groups a significant increase in the number of dead astrocytes, identified by co-labeling with the astrocytic marker GFAP and the cell death marker PI (Fig 1E amd 1F). Assessment of the number of dead astrocytes per counting region in the CA1(Fig 1G and 1H) had high interobserver agreement with the CCC of 0.9595. Quantitative data revealed a statistically significant increase in the number of dead astrocytes in the low- and high-blast compared to sham controls (P < 0.05) (Fig 1H). There was no statistically significant difference between the low- and high-blast groups; however there was a trend towards increased death of astrocytes in the high-blast group.

### Infrequent astrocytic apoptosis in the early phase of blast injury

**Fig 2:**
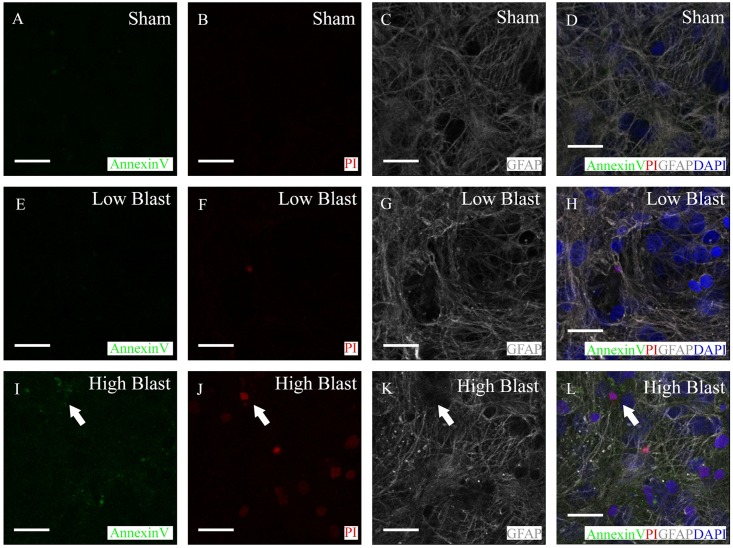
Limited early apoptotic death of astrocytes following blast exposure. At 2 h following injury, Annexin V conjugated to Alexa 488 (green; A, E, I) was used to identify apoptotic cells in sham control (A-D), low-blast (E-H), and high-blast (I-L) groups. Samples were additionally labeled with the cell death marker PI (red; B, F, J), an antibody against GFAP (gray; C, G, K), and DAPI (blue). Overlay of Annexin V, PI, GFAP, and DAPI staining (D, H, L). Annexin V positive cells (arrow) were infrequent in all three experimental groups. Almost none of the observed Annexin V positive cells were co-labeled with GFAP. Scale bars 20 μm.

Staining with Alexa 488 conjugated Annexin V identified a small number of apoptotic cells in OHCs at 2 h following sham-, low-, and high-blast injury (Fig 2). In addition, almost none of the apoptotic, Annexin V positive cells were co-labeled with astrocytic marker GFAP (Fig 2).

### Calpain mediated GFAP degradation in response to blast overpressure

**Fig 3:**
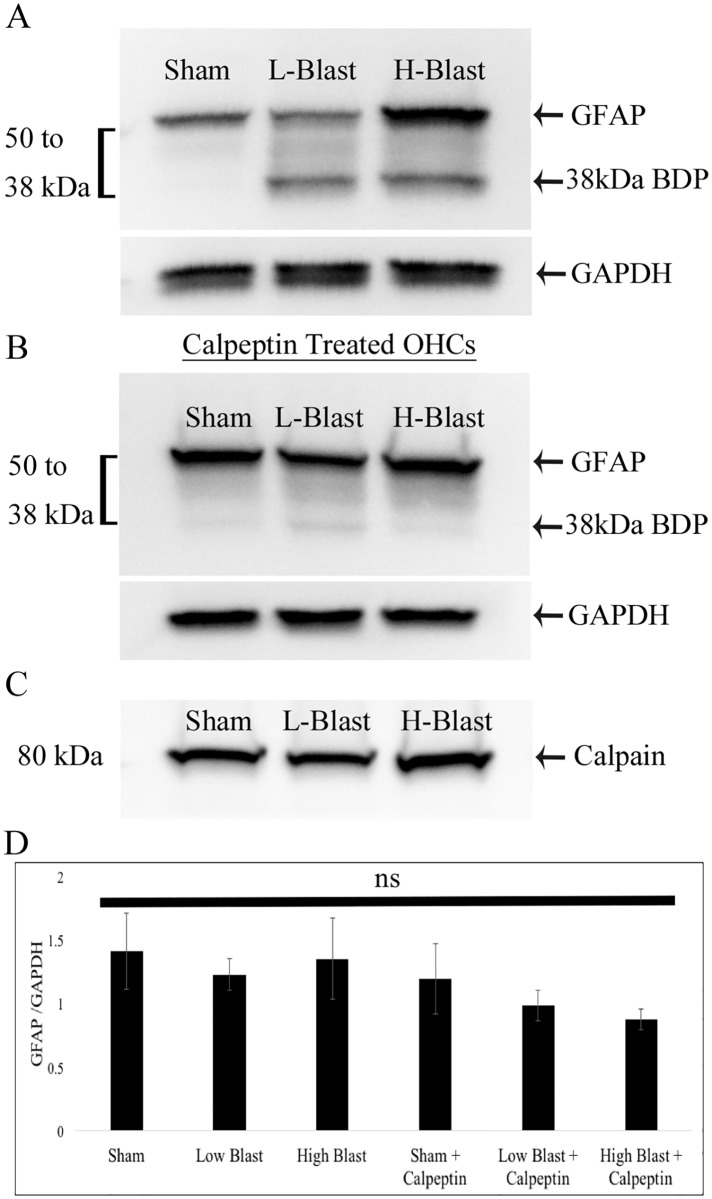
Calpain-mediated degradation of GFAP at 2 h following blast injury. Proteins were isolated from sham control (Sham), low-blast (L-Blast), and high-blast (H-Blast) OHCs at 2 h post-injury and analyzed via western blot for expression of GFAP (A, B) or calpain (C). (A) The 38 kDa calpain associated GFAP-BDP was present in blast-injured OHCs, but not in corresponding sham control OHCs. (B) Following inhibition of calpain via calpeptin treatment, this GFAP-BDP at 38 kDa was not observed. (C) Calpain expression in OHCs exposed to blast overpressure compared to sham controls was not changed at this time point. (D) Densitometry analysis of GFAP 50 kDa/GAPDH ratio for 3 independent experiments revealed no significant (ns) differences among control and blast-exposed OHCs without or with calpeptin treatment.

Results from western blot analysis of both low- and high-blast groups at 2 h following injury revealed the presence of the full length astrocytic protein GFAP (50 kDa) and a range of GFAP break down products (GFAP-BDPs) down to 38 kDa (Fig 3A). At the same time point, only the full length GFAP (50 kDa) was observed in sham control OHCs (Fig 3A). Prevalently, expression of the calpain mediated 38 kDa GFAP-BDP [109] was increased in both the low- and high- blast overpressure exposure groups (Fig 3A). Moreover, treatment with the calpain inhibitor calpeptin prevented the generation of the 38 kDa GFAP-BDP in OHCs at 2 h following blast injury (Fig 3B). However, western blot analyses revealed similar expression levels of calpain in all experimental groups (Fig 3C). In addition, densitometry analysis demonstrated that expression levels of full length GFAP were not significantly different among experimental groups with or withouth calpeptin treatment (Fig 3D).

### Acute astrocytic membrane disruption following blast exposure

**Fig 4:**
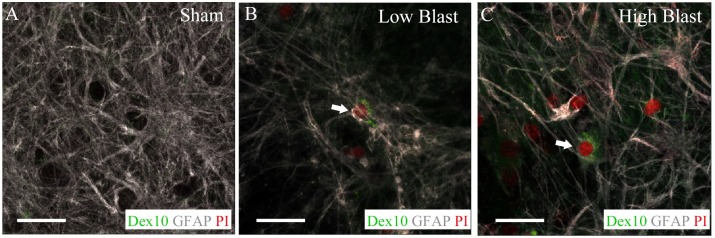
Increased astrocytic membrane permeability at 2 h post injury. Dex10 (green) and PI (red) labeled sham control (A), low-blast (B), or high-blast (C) OHCs were fixed 2 h following injury and further stained with an anti-GFAP antibody (gray). Dead astrocytes with increased membrane permeability, identified by overlap of GFAP, PI, and Dex10 staining (arrows), were only present in the low-blast (B) and high-blast (C) OHCs but not in the sham controls (A). Scale bars 20 μm.

The effect of a blast overpressure on membrane permeability was studied in OHCs at 2 h following injury using a Dex10 conjugated to Alexa 488 infusion tracer [107, 108]. Additionaly, Dex10 treated OHCs were co-labeled with the cell death marker PI and the astrocytic marker GFAP (Fig 4). We have observed an increase in the number of dextran positive cells at 2 h following injury in the low- and high-blast groups compared to the sham controls (Fig 4). Moreover, a fraction of Dex10 positive cells co-expressed PI and GFAP at 2 h following blast injury (Fig 4).

### Acute demise of GLT-1-expressing astrocytes

**Fig 5:**
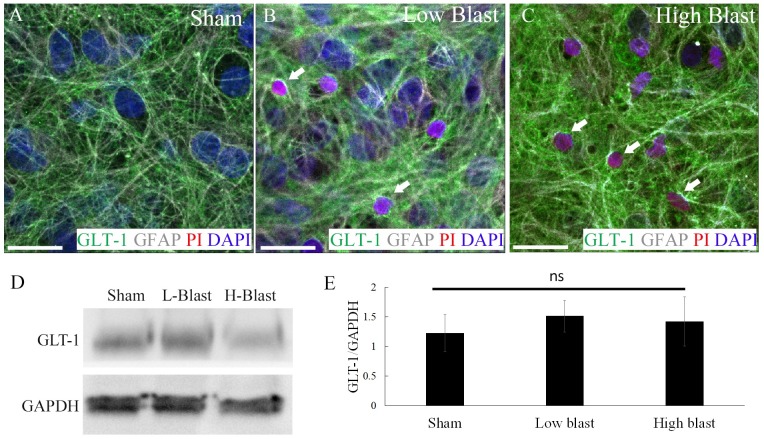
Blast-induced loss of GLT-1-expressing astrocytes. At 2 h following injury, sham control (A), low-blast (B), and high-blast (C) OHCs were stained using antibodies against GLT-1 (green), GFAP (gray), PI (red), and DAPI (blue). Dead astrocytes, identified by co-labeling of GFAP and PI (arrows) were also positive for GLT-1. (D) Representative immunoblot analyses of GLT-1 protein expression in sham control (Sham) and OHCs exposed to blast overpressure (L-Blast and H-Blast). (E) Densitometry analysis of GLT-1/GAPDH ratio for 3 independent experiments revealed no significant (ns) differences between sham control, low-blast, and high blast groups. Scale bars 20 μm.

Double labeling for GFAP and GLT-1 demonstrated significant loss of GLT-1 expressing astrocytes (Fig 5A–1C), implying decreased glutamate uptake following bTBI. However, western blot assay for GLT-1 expression levels (Fig 5D) followed by densitometry analyses (Fig 5E), did not demonstrate significant differences between sham control, low-, and high-blast OHCs at 2 h post-injury.

## Discussion

Given the recent rise in the incidence of bTBI [1–4], it is paramount to elucidate mechanisms underlying blast-evoked cell death and develop effective neuroprotective interventions. This study determined acute effects of blast exposure on astrocytes and depicted mechanisms of blast-evoked astrocytic death in an *in vitro* bTBI model. We demonstrated that blast exposure has a profound effect on astrocytes acutely following injury in both the low- and high-blast groups compared to sham controls. Our main findings included a statistically significant increase in necrotic astrocytic death in both low- and high-blast groups compared to sham controls. Additionally, our data suggest calpain mediated GFAP breakdown and increased plasmalemmal permeability as mechanisms of blast-evoked astrocytic death, and further implies glutamate dysregulation following bTBI.

We previously validated an OHC-based *in vitro* bTBI model as a tool to study cellular and molecular changes following blast exposure [34]. Advantages of OHCs cultures include preservation of 3D tissue-specific cytoarchitecture, neuronal-glial interactions, as well as interregional neuronal connectivity of an *in vivo* hippocampus [83, 93, 110]. One potential limitation is that OHCs are typically prepared from postnatal donors, as these cultures survive better [93, 95, 111]. However, OHCs prepared from neonatal donors gain more mature phenotype over the first few weeks of culture [95, 112, 113] and display similar gene regulation, protein expression, and synaptic activity of the adult hippocampus [111, 114, 115]. We cannot exclude that due to the absence of active circulatory system, bones of the scull, inputs from extra hippocampal brain regions, and systemic response in our *in vitro* bTBI model, cellular response and it’s temporal profile could be somewhat different compared to *in vivo* situation. However, previous studies demonstrated that primary and secondary cell death, as well as temporal course of damage and changes in cell death genes in OHCs exposed to mechanical or ischemic injury are similar to those observed *in vivo* [84, 116–118]. Despite limitations, there are clear benefits to the OHC reductionism and they have been extensively used to model blast and non-blast TBI [32, 33, 84, 85, 118–120].

To generate *in vitro* bTBI model, as in our previous studies [34], culture dishes with OHCs were placed outside of the shock tube and positioned 55° off axis to avoid effects of exhaust gases resulting in complex shock waves [121, 122]. OHCs were exposed to blast overpressures of approximately 150 and 280 kPa, that in our previous *in vitro* study evoked significant cell death [34]. Experimental designs similar to ours were implemented in several recent *in vitro* [32, 33, 119, 120, 123, 124] and *in vivo* bTBI studies [19, 122, 125, 126]. Notably, our shock tube model for rats [16, 28, 127] is quite similar to the model presented in this study, including design, peak overpressure and positive duration. Using the present design, it should be noted that this loading condition can be a combination of primary (shock wave overpressure) and possible tertiary loading due to acoustic impedance mismatch between the different materials (e.g., air, well, medium) that may have resulted in inertial loading that may have led to mechanical deformation (i.e., strain) of the tissue sample [34].

In the present study we analyzed cellular changes in OHCs at 2 h following blast exposure, as under the same conditions we previously observed dramatic increase in cell death from 0 to 2 h post-injury [34]. At 2 h post-injury, in addition to dead astrocytes we also observed dead neurons and microglial cells (data not shown). However, we focused our analyses on the vigorous early astrocytic response to blast overpressure that was not previously reported. In our earlier studies we demonstrated robust activation of astrocytes at 72 h post injury, however at that time point we detected only a small number of dead astrocytes [34]. Though, corresponding to our current results, we observed dead microglial cells at 4 and 24 h post-injury [34]. Collectively, results from our present and previous studies show acute astrocytic demise followed by robust activation at 72 h post-injury. Similar to our data, astrocytic damage and PI uptake was observed in the stretch-injury model only immediately following injury, but not at the 24 and 48 h post-injury [128, 129]. Correspondingly, results collected from human blunt TBI cases implied that number of astrocytes initially decreases within the first 24 h post-injury and then again increases at the later time points, indicating formation of reactive gliosis [76]. However, significant changes in viability of astrocytes were not observed in astrocyte monocultures exposed to the blast overpressure [130, 131]. Assessments were conducted only at later time points post-injury, which could explain why initial astrocytic susceptibility was not observed. Moreover, blast shock wave may not have the same biological effects in astrocyte monocultures as in OHCs that more closely resemble *in vivo* situation [83, 93, 110]. In agreement with our data, death of astrocytes [67, 78] and their activation [35, 66, 67, 125, 132, 133] were detected in animal studies following shock wave exposure. On the contrary, several *in vivo* bTBI studies did not observe increased GFAP expression [134–136], which could be due to the different experimental conditions and assessment timing.

In this study we observed significant co-labeling of GFAP-expressing astrocytes with the cell death marker PI, which identified necrosis as the primary mechanism of astrocytic death in OHCs at 2 h following blast exposure. Additionally, we studied presence of apoptotic astrocytes in the acute phase of bTBI using co-labeling with Annexin V and GFAP. Fluorescently labeled Annexin V was previously used in OHCs and different *in vivo* models of brain injury [137–139] to detect apoptotic cells even within several hours following injury [140–142]. The limited detection of Annexin V positive cells at 2 h following blast exposure, rules out apoptosis as a significant contributing factor to astrocytic death acutely following blast exposure. It was previously shown that both necrotic and apoptotic cell death are implicated in bTBI [133, 143–145]. Studies in monkey [67] and rat [78] bTBI models detected apoptotic astrocytes at 1 month and 1 day post-injury, respectively. However, those studies did not assess astrocytic apoptotic death at more acute time points as we did. Therefore, our data together with results collected in *in vivo* bTBI models [67, 78] imply that apoptosis may play a significant role in astrocytic death at later time points.

Beside the acute death, we observed blast-evoked shearing and morphological changes in astrocytes. Clasmatodendrosis, an irreversible astroglial degeneration characterized by beading and dissolution of their processes [146], was also observed at 2 h following injury. In accordance with our results, other groups demonstrated that shock waves can generate shear forces in the brain tissue [25, 132, 147] and induce elongation and deformation of the cell organelles [25, 145, 148]. Trauma-induced morphological changes of astrocytes, such as swelling and ultrastructural alterations, were previously perceived in an *in vitro* model of fluid percussion injury [149, 150], stretched-injured astrocytes [151], a rat bTBI model [152], and in patients with cerebral contusions [77, 153]. Likewise, clasmatodendrosis of astrocytes was detected in TBI patients from 1 h up to 14 days post injury [154]. Clasmatodendrosis in astrocytes was linked with the autophagic cell death [155, 156], suggesting autophagy as an additional mechanism of astrocytic death in OHCs at 2 h following blast exposure. Accordingly, an increase in autophagy that can lead to cell death was previously observed following blunt TBI [157–160] and bTBI [23].

Furthermore, our study revealed cleavage of GFAP by calpain as a potential mechanism of early astrocytic death, based on the presence of calpain-mediated 38 kDa GFAP-BDP at 2 h following injury. Moreover, when we introduced the calpain inhibitor calpeptin into the OHCs culture medium, formation of the 38 kDa GFAP-BDP in the blast-injured groups was prevented, while expression levels of full length GFAP remained similar. Expression levels of calpain were similar in all experimental groups, suggesting only increase in calpain activity following blast exposure due to the calcium overload [161]. Previous human and animal studies established that calpains play a key role in neuropathological changes following blunt TBI and that application of calpain-inhibitors can have protective effects [109, 162–164]. Recent studies in a mouse bTBI model also reported increased calpain-mediated cytoskeletal breakdown [165, 166]. Corresponding to our data, it has been shown that beside neuronal proteins, GFAP can be calpain substrate as well [109, 167]. In addition, it has been shown that both increased serum levels of GFAP [168–170] and GFAP-BDPs [109, 171–173] are strong biomarkers of brain injury.

The overlap of Dex10, GFAP, and PI staining observed in the present study in low-and high-blast groups, indicated plasmalemmal disruption as an additional mechanism of astrocytic death in response to the blast overpressure at 2 h following injury. In addition, a fraction of Dex10-labeled cells in OHCs exposed to blast overpressure did not express GFAP, implying that blast exposure also causes rapid changes in cell membrane permeability in other cell types in OHCs. Complex changes in cell membrane permeability that can lead to the ionic imbalances and activation of several cellular pathways have been previously described in stretched-injured astrocytes [129, 174, 175], as well as in blunt and diffuse TBI models [107, 108, 176, 177]. In addition, increased cell permeability and cytoskeletal damage has been observed following blast exposure in the dorsal root ganglion (DRG) [178] and SH-SY5Y human neuroblastoma cells [31]. Correspondingly, acute decrease in GFAP/Tau was detected in the mouse brain after blast exposure, which most likely was result of blast-induced perturbation of neuronal and astrocytic cell membranes and protein leakage across the disrupted blood-brain barrier [179].

Based on the essential role of astrocytes in maintaining brain homeostasis, it is feasible that the acute death of astrocytes observed in our studies could be implicated in secondary neuronal loss following bTBI. A link between acute astrocytic death and delayed neuronal loss has also been postulated in models of ischemic and blunt brain injury [59, 74]. Despite no apparent changes in GLT-1 protein expression, significant loss of GLT-1 expressing astrocytes observed in our studies at 2 h following blast injury, implies a reduction of glutamate uptake that can further cause excitotoxic neuronl and glial death [180–183]. Under normal physiological conditions, astrocytes regulate extracellular glutamate concentration through release and transport via glutamate transporters [37, 54, 184, 185]. Out of five sodium-dependent glutamate transporters that have been cloned [185], GLT-1 is responsible for the majority of glutamate uptake [186, 187] and it is mainly expressed by astrocytes [188]. However, neurons and microglial cells also express GLT-1 [75, 188, 189] and it is feasible that blast-evoked loss of these cells could be also implicated in the disruption of glutamate uptake. In addition, previous studies demonstrated that both excessive glutamate release from neurons and dysfunction of astrocytes could contribute to a prompt increase of extracellular glutamate concentration following TBI [182, 190, 191]. Likewise, several studies reported decrease in GLT-1 expression in blunt TBI models [75, 192, 193] and in human TBI [76]. In our previous studies we have demonstrated the spread of cell death following bTBI and that at 72 h post-injury majority of dead cells were neurons [34]. Based on data from this study, we speculate that early demise of astrocytes and potential glutamate dysregulation could aggravate neuronal loss at the later time points post-injury.

In conclusion, this study demonstrated substantial acute effects of blast overpressure on astrocytes in OHCs. Understanding the damage incurred by astrocytes at 2 h post-injury in this *in vitro* model paves a path for understanding the role of astrocytes in bTBI neuropathology. Results presented here provide steps toward future research, which will examine whether the acute astrocytic demise significantly contributes to the delayed neuronal loss and whether astrocytes could be targeted to prevent spread of neurodegeneration following bTBI.
